# Direct evidence for BBSome-associated intraflagellar transport reveals distinct properties of native mammalian cilia

**DOI:** 10.1038/ncomms6813

**Published:** 2014-12-15

**Authors:** Corey L. Williams, Jeremy C. McIntyre, Stephen R. Norris, Paul M. Jenkins, Lian Zhang, Qinglin Pei, Kristen Verhey, Jeffrey R. Martens

**Affiliations:** 1 Department of Pharmacology and Therapeutics, College of Medicine, University of Florida, 1200 Newell Drive, PO Box 100267, Gainesville, Florida 32610, USA; 2 Department of Cell and Developmental Biology, University of Michigan, 109 Zina Pitcher Place, 3041 Biomedical Science Research Building (BSRB), Ann Arbor, Michigan 48109, USA; 3 Department of Pharmacology, University of Michigan Medical School, 1301 MSRB III, 1150 West Medical Center Drive, Ann Arbor, Michigan 48109-5632, USA; 4 Department of Biostatistics, University of Florida, RM5225, 2004 Mowry Road, Gainesville, Florida 32611, USA

## Abstract

Cilia dysfunction underlies a class of human diseases with variable penetrance in different organ systems. Across eukaryotes, intraflagellar transport (IFT) facilitates cilia biogenesis and cargo trafficking, but our understanding of mammalian IFT is insufficient. Here we perform live analysis of cilia ultrastructure, composition and cargo transport in native mammalian tissue using olfactory sensory neurons. Proximal and distal axonemes of these neurons show no bias towards IFT kinesin-2 choice, and Kif17 homodimer is dispensable for distal segment IFT. We identify Bardet–Biedl syndrome proteins (BBSome) as *bona fide* constituents of IFT in olfactory sensory neurons, and show that they exist in 1:1 stoichiometry with IFT particles. Conversely, subpopulations of peripheral membrane proteins, as well as transmembrane olfactory signalling pathway components, are capable of IFT but with significantly less frequency and/or duration. Our results yield a model for IFT and cargo trafficking in native mammalian cilia and may explain the penetrance of specific ciliopathy phenotypes in olfactory neurons.

## Abstract (web summary)

Loss of olfactory function is one of the many symptoms of the ciliopathy Bardet–Biedl syndrome. Williams *et al*. show that Bardet–Biedl proteins are components of intraflagellar transport particles within cilia, and directly visualize their trafficking in native mammalian olfactory neurons.

## Graphical abstract

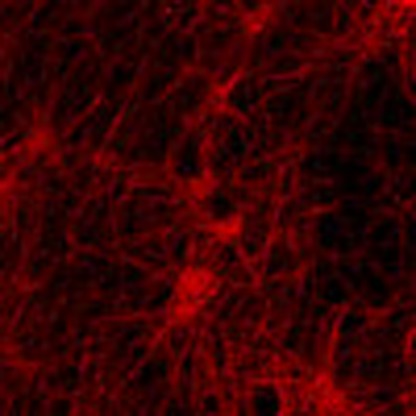

The cilium is a sensory organelle that serves specialized roles on diverse cell types throughout eukaryotes. Disruption of cilia in vertebrates gives rise to developmental patterning defects, progressive degenerative disorders and sensory deficits. Penetrance of congenic human ciliopathy disease phenotypes varies among different tissues and can be influenced by the gene that is disrupted, the nature of mutation and the genetic background of the individual^1^. The precise mechanisms of this variable penetrance are unclear, in particular when mutations affect ubiquitously expressed cilia genes. Across eukaryotes, cilia and flagella are built and maintained by intraflagellar transport (IFT), a process in which macromolecular protein ‘trains’ comprising IFT-A and IFT-B subcomplexes bidirectionally traverse the ciliary microtubule axoneme via association with kinesin and dynein motors^2^. Mutations in several human IFT genes are linked to a group of gestational skeletal disorders^3^,^4^,^5^,^6^ and, recently, one gene encoding a core IFT complex B protein was revealed as a disease locus for Bardet–Biedl syndrome (BBS)^7^. BBS is a heterogeneous pleiotropic ciliopathy that shows penetrance in a number of organs and clinically manifests in obesity, polydactyly, renal cyst formation, retinal cell death, male infertility and anosmia. Of note, only these latter two conditions are due to the absence of cilia structures^8^,^9^,^10^ and it is unclear why cilia in one cell type or tissue are lost but persist (or degenerate slowly) in other organs.

Work in lower eukaryotes, namely in *Chlamydomonas reinhardtii* and *Caenorhabditis elegans*, has demonstrated functional links between the IFT machinery and BBSome in cilia formation and maintenance and, moreover, currently accounts for the majority of our knowledge concerning IFT dynamics^11^,^12^. In mammals, IFT has been described in only a small number of studies on cultured cells^13^,^14^,^15^,^16^,^17^,^18^ and the fundamental questions of how the mammalian IFT machinery operates in native cilia or whether it associates with BBS proteins have not been addressed. These questions are particularly relevant in the context of IFT gene mutations causing BBS^7^ and BBS disease penetrance within the olfactory system where BBS protein disruption is detrimental to ciliation and odour detection^9^,^10^. Importantly, these unanswered questions can have significant implications for therapeutic strategies to treat ciliopathies in different organ systems.

IFT studies in vertebrates present a challenge, because most cilia of vertebrate tissues are inaccessible in the live animal. In contrast, olfactory cilia are uniquely amenable to live analysis due to their direct contact with the external environment via the nasal passage. This enables manipulation of live olfactory sensory neurons (OSNs) without disrupting synaptic connections. Here we exploited adenovirus (AV)-mediated protein expression and live-cell time-lapse imaging to capture IFT and cargo-trafficking events within native mammalian olfactory cilia. We find that the IFT machinery in OSN cilia employs two kinesin motors (heterotrimeric kinesin-2 and homodimeric Kif17), but Kif17 is not required for IFT or OSN cilia maintenance. OSN IFT particle velocities vary stochastically among neurons, but velocities in different cilia of individual neurons are similar to one another. We reveal constituents of the core BBS protein complex as participants in OSN IFT that exist in 1:1 stoichiometry with IFT particles. In addition, peripheral membrane Arl proteins and polytopic transmembrane signalling proteins traffic with IFT in OSN cilia, but the transmembrane proteins do so much less frequency and/or duration. Overall, our findings provide framework for the first model of IFT in a native mammalian setting and implicate the relationship between IFT and the BBSome as a likely culprit in the penetrance of BBS mutations on ciliation in the olfactory epithelium (OE).

## Results

### Distinct properties of the OSN ciliary axoneme and membrane

**Figure 1:**
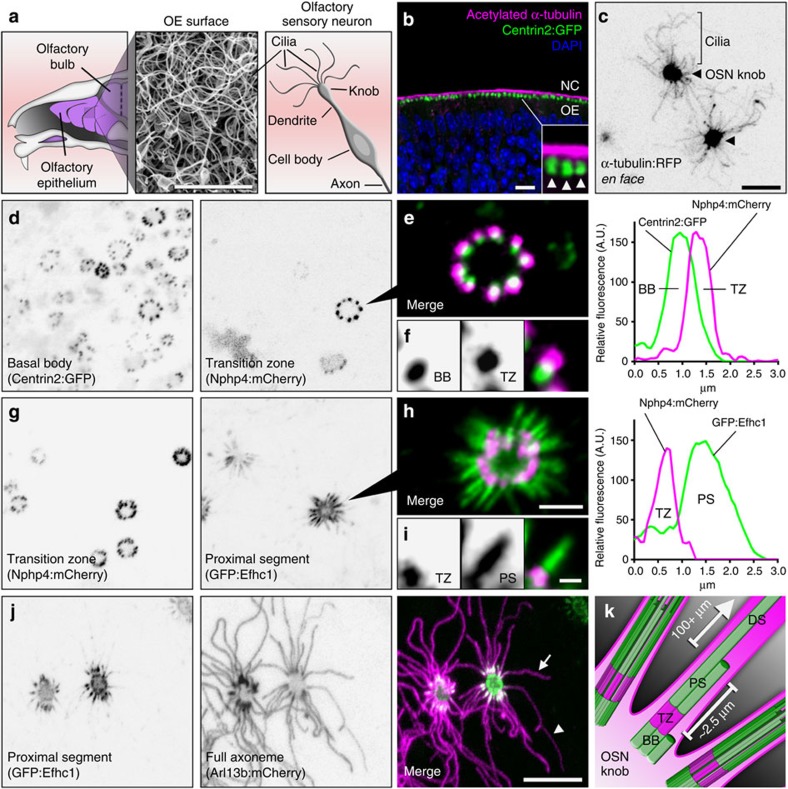
Molecular architecture of OSN cilia. (**a**) (Left) Illustration depicting a sagittal view of the mouse OE. (Middle) Scanning electron micrograph of the OE surface showing a dense mat of olfactory cilia. (Right) Illustration depicting multiple cilia on an OSN. (**b**) Representative confocal image of a coronal OE section from a Centrin2:GFP transgenic mouse stained for acetylated α-tubulin to reveal cilia. NC, nasal cavity. (Arrowheads) Side-by-side dendritic knobs revealed by Centrin2:GFP signal. (**c**–**j**) Representative live *en face* confocal images of AV-transduced native OE expressing various cilia domain-specific marker proteins. (**c**) AV-α-tubulin:RFP expression reveals many individual OSN microtubule axonemes projecting from two OSN dendritic knobs (arrowheads) on the surface of the OE. (**d**) Centrin-2:GFP transgenic mouse transduced with AV-Nphp4:mCherry. From left: Centrin-2:GFP-labelled basal bodies (BBs) line the periphery of OSN knobs. Nphp-4:mCherry marks ciliary transition zones (TZ), distally associated with each BB as seen in **e**. (**f**) A single TZ/BB unit. Far right: representative line-scan intensity plot showing the fluorescence profile of a single BB/TZ. (**g**) Co-expression of AV-Nphp-4:mCherry and doublet microtubule marker AV-GFP:Efhc1. GFP:Efhc1 is restricted to the proximal segment (PS) of each OSN ciliary axoneme. (**i**) A single TZ/PS unit. Far right: representative line-scan intensity plot showing the fluorescence profile of a single TZ/PS. (**j**) Co-expression of AV-GFP:Efhc1 and cilia peripheral membrane marker AV-Arl13b:mCherry. Arl13b:mCherry reveals axonemes of variable length (compare arrow and arrowhead), each extending from proximal segments marked by GFP:Efhc1. (**k**) Illustration depicting the ultrastructure of an OSN cilium in which a basal body gives rise to a transition zone followed by a ~2.5 μm doublet microtubule proximal segment and finally a singlet microtubule distal segment (DS) of variable length, sometimes exceeding 100 μm. Scale bars, 5 μm (**a**); 10 μm (**b**–**d**,**g**,**j**); 2.5 μm (**e**,**h**); 1.25 μm (**f**,**i**).

To properly dissect ciliary trafficking dynamics in native mammalian olfactory cilia, we first delineated the organization of OSN cilia architecture via molecular profiling in living mouse tissue. In addition to allowing measurement of cilia dynamics, live analysis of ultrastructural features enables accurate appraisal of cilia properties that can be lost with fixation. We used AV-mediated ectopic expression of fluorescent protein (FP)-tagged proteins that comprise various structural elements of the OSN cilium (Fig. 1). *En face* microscopic investigation of red FP-tagged α-tubulin revealed the microtubule backbones of the many cilia emanating from the dendritic knob of each AV-transduced OSN (Fig. 1c). OSN ciliary basal bodies and transition zones were identified using transgenic Centrin2:GFP mice ectopically expressing Nphp4:mCherry (Fig. 1d–f). Based on Nphp4:mCherry fluorescence, OSN ciliary transition zones measure 0.95±0.12 μm in length (*n*=72 cilia, 9 OSNs, 3 mice), similar to frog and nematode^19^,^20^. To visualize OSN axoneme microtubule organization, we used green FP (GFP)-tagged EF-hand domain containing 1 (GFP:Efhc1), which associates with interdoublet links connecting doublet microtubules^21^ and is highly enriched in OSNs^22^. GFP:Efhc1 was restricted specifically within the proximal-most region of OSN cilia, revealing the OSN doublet–singlet microtubule transition at 2.34±0.42 μm from the base of the transition zone (Fig. 1g–i, *n*=141 cilia, 11 OSNs, 3 mice). Finally, to differentiate the proximal versus distal segments of OSN cilia^23^, we co-expressed GFP:Efhc1 with mCherry-tagged ADP-ribosylation factor-like 13b (Arl13b:mCherry), which localizes along the full length of OSN cilia^6^ (Fig. 1j). Collectively, our data showing highly restricted localization patterns of FPs with strong correlation to previous electron microscopy data in rodent^23^ demonstrate that ectopic expression accurately delineates endogenous protein localization in cilia of live OSNs.

**Figure 2:**
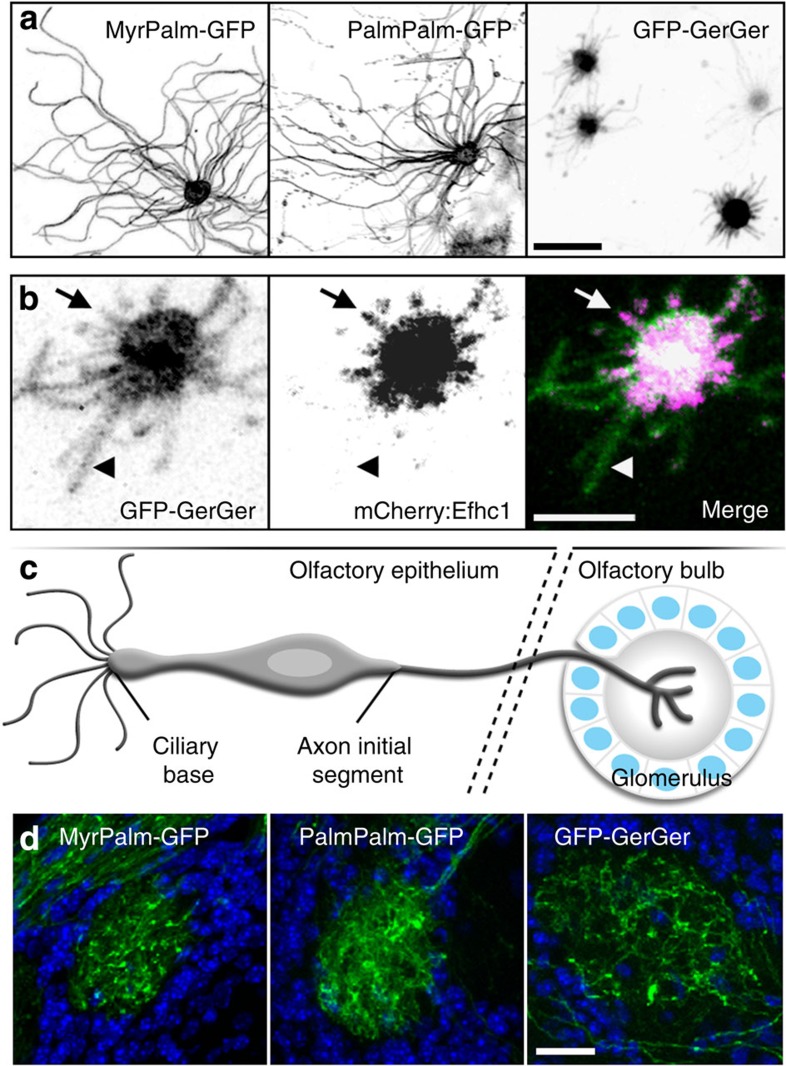
Differential membrane enrichment of acyl and prenyl anchors in OSN ciliary subdomains. (**a**) Representative confocal *en face* images captured from whole OE transduced with AV- (left) MyrPalm-GFP, (middle) PalmPalm-GFP or (right) GFP-GerGer. Whereas MyrPalm and PalmPalm are present throughout OSN ciliary membranes, GerGer is restricted to within the proximal regions of cilia near the OSN knobs. (**b**) GerGer-GFP ciliary membrane localization can extend beyond the proximal doublet microtubule segments as revealed by AV-mCherry:Efhc1 expression. Arrows highlight a cilium where GFP-GerGer localization is coincident with mCherry:Efhc1. Arrowheads point to a ciliary segment where GFP-GerGer localizes beyond mCherry:Efhc1. (**c**) Illustration of an OSN depicting the location of the axon initial segment and of synaptic termini in a glomerulus of the olfactory bulb. (**d**) Lipid-anchored GFPs freely access OSN axons. Representative confocal images of fixed sections through glomeruli of the olfactory bulb from mice expressing (left) MyrPalm-GFP, (middle) PalmPalm-GFP and (right) GFP-GerGer are shown. Empty circular regions surrounded by DAPI (4',6-diamidino-2-phenylindole; blue)-labelled nuclei represent glomeruli where OSN axons terminate. Scale bars, 10 μm (**a**); 5 μm (**b**); 20 μm (**d**).

Specific lipid modifications are sufficient to sequester proteins in membrane microdomains of unique lipid composition or organization^24^. This is particularly relevant to ciliary membrane organization, given that loss of lipid modifications is detrimental to ciliary localization of Arl13b^30^, Retinitis Pigmentosa 2 (refs 25, 26), cystin^27^ and fibrocystin^28^. We compared the ability of myristoylation and palmitoylation (MyrPalm), dual palmitoylation (PalmPalm) and geranylgeranyl (GerGer) signals (which differentially partition into detergent-resistant membrane domains (Supplementary Fig. 1a,b, see Supplementary Methods)), to enrich GFP within the OSN ciliary membrane. MyrPalm-GFP and PalmPalm-GFP were present along the length of olfactory cilia (Fig. 2a); however, GFP-GerGer was constrained within the OSN ciliary proximal region (Fig. 2a). GFP-GerGer localization extended beyond mCherry:Efhc1 (Fig. 2b), indicating that the GerGer membrane microdomain is distinct from and more extensive than the membrane surrounding the OSN cilium proximal segment proper. In contrast to the differential lipid partitioning observed in OSN cilia, we found that OSN axons are equally permissive to each of the three lipid modifications as seen via GFP infiltration of olfactory bulbs (Fig. 2d). Although the axon initial segment^29^ and ciliary base^30^,^31^,^32^,^33^,^34^,^35^,^36^ both act as a filter for specific transmembrane or membrane-associated proteins, our data show both are permissive to the lateral enrichment of lipid modified proteins. However, beyond the ciliary base, there exists a differential partitioning of lipid anchors between the OSN proximal and distal segments.

Finally, to measure OSN cilia number and length, we used MyrPalm-GFP as an inert marker. OSNs possessed 13±5 cilia (*n*=81 OSNs, 3 mice) with lengths ranging from 2.5 to 110 μm (22.0±12.8 μm average, *n*=1,083 cilia, 3 mice; Supplementary Fig. 2a). We saw no correlation between the number and length of cilia on a given cell (Supplementary Fig. 2b; Pearson’s coefficient *R*^2^=0.04829) and cilia lengths were highly variable within cells, indicating that OSN cilia length varies stochastically.

### Multiple kinesin-2 subtypes traverse entire OSN axonemes

**Figure 3:**
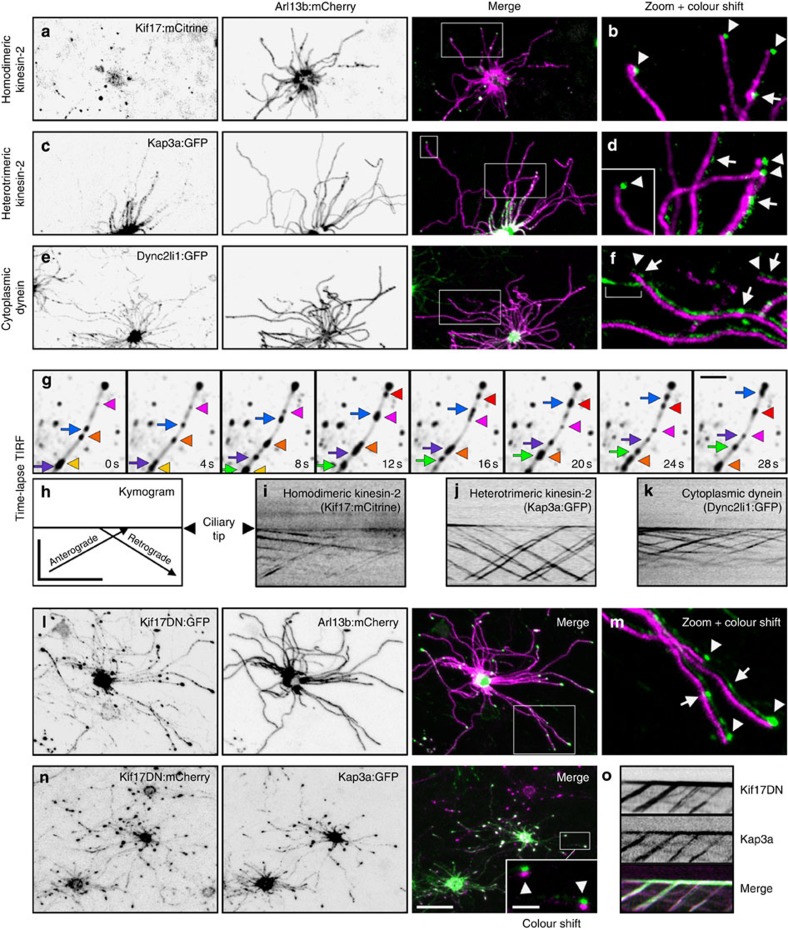
Homodimeric and heterotrimeric kinesin-2 motors cooperatively traffic along the full length of OSN cilia. (**a**–**c**) Representative live *en face* confocal images of native OE ectopically expressing components of the IFT motor complexes. (**a**) Homodimeric kinesin-2 protein Kif17:mCitrine is present in cilia and at ciliary distal tips. (**b**) Accumulations of Kif17:mCitrine at distal tips (arrowheads) and in puncta along axonemes (arrow). (**c**) Heterotrimeric kinesin-2 component Kap3a:GFP is abundant along cilia axonemes. (**d**) Accumulations of Kap3a:GFP at ciliary distal tips (arrowheads) and distribution along axonemes (arrows). (**e**) Cytoplasmic dynein component Dync2li1:GFP is detectable along cilia axonemes. (**f**) Dync2li1:GFP is highly abundant along OSN cilia, detectable as discrete puncta (arrows), and is present at ciliary distal tips (arrowheads). Bracket indicates signal in a cilium from an OSN expressing only AV-Dync2li1:GFP. (**g**) TIRFm time-lapse capture of Kap3a:GFP particle movement in an OSN cilium. Exposures from different time points were overlayed onto an average intensity projection of the entire series to resolve individual particles in relation to the axoneme (ciliary tip is to the right, top). (Arrows) Anterograde particles. (Arrowheads) Retrograde particles. (**h**–**k**) Line-scan kymograms generated from single cilia of OSNs ectopically expressing the indicated motor component. (**h**) Orientation of particle movement in relation to the ciliary tip. Axes represent distance (μm) over time (s). (**i**) Kif17:mCitrine-, (**j**) Kap3a:GFP- and (**k**) Dync2li1:GFP-labelled particles moving in anterograde and retrograde directions. (**l**) Disruption of Kif17 is not detrimental to the maintenance of OSN axonemes. Representative live *en face* confocal image of an OSN co-expressing AV-Kif17DN:GFP and AV-Arl13b:mCherry. (**m**) The presence of Kif17DN:GFP at distal tips (arrowheads) and in puncta along axonemes (arrows). (**n**,**o**) Kif17DN is transported to distal tips in IFT particles containing Kap3a. (**n**) Representative live *en face* confocal image of an OSN co-expressing AV-Kif17DN:mCherry and AV-Kap3a:GFP shows their co-localization in cilia and at ciliary distal tips (arrowheads). (**o**) Kymogram generated from a cilium of an OSN expressing (top) AV-Kif17DN:GFP and (middle) AV-Kap3a:mCherry. Scale bars, 10 μm (**a**–**e**,**l**,**n**); 2.5 μm (zoomed panels); 2.5 μm (**g**); 10 μm × 30 s (**h**–**k**,**o**).

Our data highlight structural and membrane compositional aspects distinguishing specific subdomains of OSN cilia, but the mechanisms by which the proximal and distal segments are established remain unclear. In *C. elegans* amphid/phasmid channel sensory cilia, two kinesin motors function in coordination to build the proximal/middle and distal ciliary axoneme segments^37^. To examine whether multiple kinesin motors are used in OSN cilia, we expressed FP-tagged homodimeric Kif17 and a component of the heterotrimeric kinesin-2 (Kap3a). Both Kap3a and Kif17 localized in puncta along the full length of OSN axonemes and showed accumulations at ciliary tips (Fig. 3a–d). Thus, heterotrimeric kinesin-2 is not restricted to the proximal segment of OSN cilia as seen in nematode amphid/phasmid channel cilia. FP-tagged Dync2li1, a component of the ciliary retrograde dynein motor complex, showed similar localization (Fig. 3e,f). To assess ciliary movement of the motor complex proteins, we employed high contrast, rapid acquisition, total internal reflection fluorescence microscopy (TIRFm) on OSNs expressing Kap3a:GFP, Kif17:mCitrine or Dync2li1:GFP. Strikingly, each protein showed robust, processive particle movement in both the anterograde and retrograde directions along the entire OSN cilia (Fig. 3g–k and Supplementary Movie 1). To our knowledge, this is the first capture of IFT in any native tissue of a vertebrate and/or mammalian system.

Our time-lapse data indicates that both heterotrimeric kinesin-2 and Kif17 participate in IFT along the entire OSN axonemes. We therefore reasoned that the presence of OSN cilia distal segments may not depend solely on the function of Kif17 as is seen with OSM-3 in *C. elegans* amphid/phasmid channel cilia^37^. To test this hypothesis, we used a truncated dominant-negative version of Kif17 (Kif17DN) lacking the motor domain. Expression of Kif17DN was not detrimental to OSN cilia formation (Fig. 3l,m), and Kif17DN showed mobility along the entire axonemes via association with IFT particles containing Kap3a (Fig. 3n,o and Supplementary Movie 2). These data indicate that distal segment formation and/or maintenance in OSN cilia is not dependent on Kif17 alone, and that heterotrimeric kinesin-2 is capable of IFT trafficking along the entire length of OSN axonemes when Kif17 is disrupted. Thus, we conclude that OSN cilia use a unique and heretofore undescribed programme featuring at least two types of kinesin motors for trafficking along distal singlet microtubules.

### Stochastic variation in IFT velocities among OSNs

**Figure 4:**
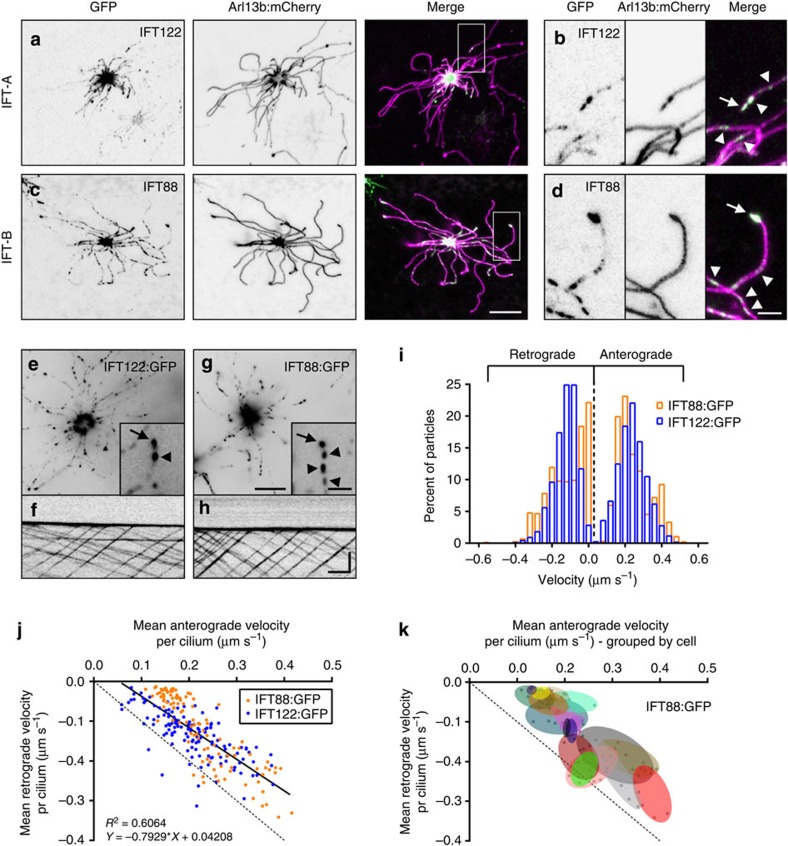
Stochastic variation in IFT velocities among OSNs. (**a**,**c**,**e**) Representative live *en face* confocal images of native OE ectopically expressing components of the IFT particle subcomplexes. Localization of (A) IFT-A subcomplex protein IFT122:GFP and (**c**) IFT-B subcomplex protein IFT88:GFP in the OSN knob and in puncta along the length of cilia as revealed by Arl13b:mCherry co-expression. Zoomed colour-shifted images show accumulation of (**b**) IFT122:GFP and (**d**) IFT88:GFP at the distal tips of cilia (arrow) and in puncta along axonemes (arrowheads). (**e**,**g**) Representative live *en face* TIRFm images captured from OSNs expressing IFT122:GFP or IFT88:GFP. Two-second exposures are shown. Insets show GFP puncta on OSN ciliary axonemes (arrowheads) and accumulations at ciliary distal tips (arrows). (**f**,**h**) Representative line-scan kymograms generated from single cilia of OSNs ectopically expressing the indicated IFT protein. (**i**) Histogram distribution of IFT122:GFP and IFT88:GFP particle velocities. IFT122:GFP, *n*=1,315 anterograde particles and 1,001 retrograde particles, 4 mice; IFT88:GFP, *n*=1,060 anterograde particles and 881 retrograde particles, 5 mice. (**j**) Plot of individual cilium mean anterograde versus retrograde velocities, showing a positive association between the magnitude of anterograde and retrograde velocities in a given cilium (Pearson’s coefficient *R*^2^=0.6064). Linear regression is shown. (**k**) Plot of individual cilium mean anterograde versus retrograde velocities, clustered by OSN (each group of points encapsulated by a coloured oval represents the cilia from a single cell). IFT88:GFP data set is shown. Scale bars, 10 μm (**a**,**c**); 2.5 μm (**b**,**d**); 10 μm, inset, 2.5 μm (**e**,**g**); 5 μm × 10 s (**f**,**h**).

The basic mechanistic principles by which IFT trains enable cargo transport within cilia are well established from work in homogeneous age-synchronous cell populations or groups of cells derived from an invariant lineage, but we have no understanding about IFT dynamics in the complex setting of a native mammalian tissue or in multiciliated cells. As individual OSNs differ from each other on many levels including odourant receptor expression, basal stimulation/activation state, age and spatial distribution, the OE is well suited for analysis of IFT dynamics in a heterogeneous context. We measured IFT velocities using FP-tagged components of the IFT-A and IFT-B subcomplexes, which undergo robust bidirectional motility along the full length of OSN cilia (Fig. 4a–h and Supplementary Movie 3). To differentiate anterograde versus retrograde trajectories, only IFT particles travelling in clear view of the ciliary tip were included in our analysis and are representative of IFT velocities specifically on the OSN distal segment. Velocities of both anterograde and retrograde IFT particles ranged from nearly stationary to approaching 0.6 μm s^−1^ (Fig. 4i), with anterograde and retrograde means of 0.22±0.08 and −0.14±0.08 μm s^−1^, respectively (*n*=2,316 IFT122:GFP-labelled particles, 1,941 IFT88:GFP-labelled particles, 300 cilia, 55 OSNs and 9 mice). In addition, the magnitude of anterograde and retrograde mean particle velocities within individual OSN cilia strongly correlate (Fig. 4j, Pearson’s coefficient *R*^2^=0.6064). Notably, we saw stochastic variation in anterograde and retrograde velocities among neurons and the velocities measured in multiple cilia within individual cells were more similar to each other than to cilia on other cells (Fig. 4k; permutation test with 20,000 iterations, *P*<0.0001 for both anterograde and retrograde velocities, see Methods) (*n*=1,941 IFT88:GFP-labelled particles, 160 cilia, 25 OSNs and 5 mice). Thus, differential regulation of IFT velocity occurs across the OSN population and the velocities of IFT particles in the many cilia projecting from a single OSN are controlled together within each cell.

Although the majority of IFT particles in OSN cilia moved with constant velocity, in some instances we observed non-processive particle trajectories (Supplementary Fig. 3). These included single-particle velocity changes (stalling/restarting and mid-cilium direction reversal), which may represent spatio-temporal events where IFT particles perform maintenance on the axoneme through delivery and/or pickup of cargo^38^. We also observed unidirectional particle interactions (fusions and fissions) and rare anterograde and retrograde particle collision/fusion events. These observations imply that individual microtubules may be simultaneously used by both unidirectional and opposing particles, and that particle remodelling is not strictly limited to events at the ciliary base and microtubule tips^39^. Finally, increased particle size was occasionally observed, which may reflect a form of IFT avalanching at the ciliary base or tip as described in *C. reinhardtii*^40^ and/or snowballing from multiple fusion events along the axoneme.

### The BBSome is a constituent of the mammalian OSN IFT complex

**Figure 5:**
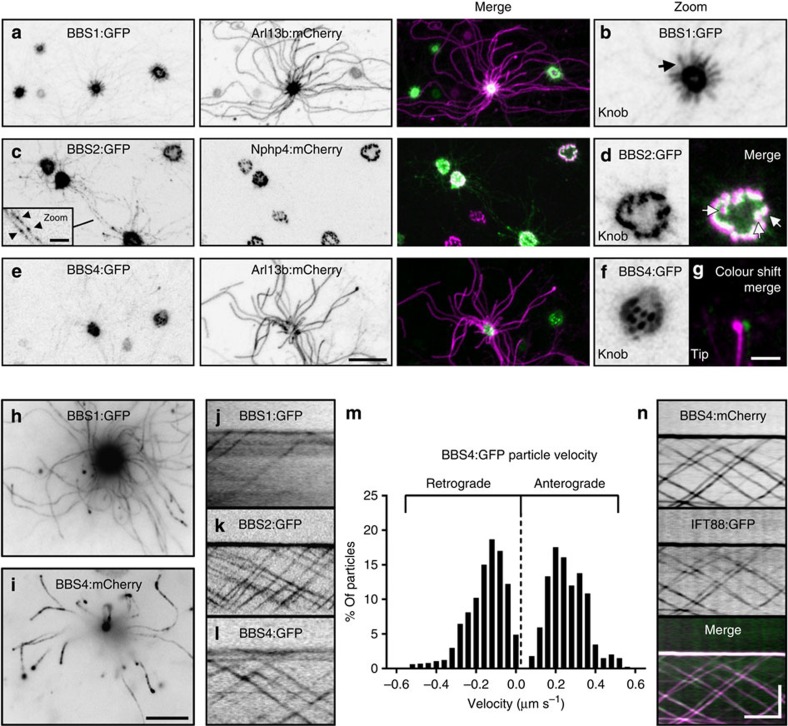
The BBSome is a constituent of OSN IFT particles. (**a**–**f**) Representative live *en face* confocal images of native OE ectopically expressing components of the BBSome. (**a**) Image of OE from animal transduced with AV-BBS1:GFP and AV-Arl13b:mCherry. BBS1:GFP concentrates strongly within the OSN knob and is distributed uniformly along the proximal-most segments (arrow) of OSN cilia as shown in the zoomed image (**a**). (**c**,**d**) Image of OE from animal transduced with AV-BBS2:GFP and AV-Nphp4:mCherry. (**c**) BBS2:GFP concentrates strongly within the OSN knob and is visible along ciliary axonemes in puncta (inset, arrowheads). (**d**) BBS2:GFP is enriched in basal bodies in the OSN knob. Merged panel shows BBS2:GFP basal body localization in close association with transition zones (arrows) as revealed by Nphp4:mCherry labelling. (**e**) Image of OE from animal transduced with AV-BBS4:GFP and AV-Arl13b:mCherry. BBS4:GFP concentrates strongly within the OSN knob. (**f**) Zoomed panel showing a transduced OSN knob where BBS4:GFP appears to localize at basal bodies. (**g**) Merged and zoomed panel shows accumulation of BBS4:GFP at the tip of an OSN cilium as revealed by Arl13b:mCherry labelling. (**h**–**n**) TIRFm uncovers BBSome protein association with IFT particles in OSN cilia. (**h**,**i**) Collapsed maximum intensity projection of TIRFm time-series images captured from OSNs ectopically expressing (**h**) AV-BBS1:GFP or (**i**) AV-BBS4:GFP. The collapsed time series show that both BBS1:GFP and BBS4:GFP are present in OSN cilia. (**j**–**l**) BBSome proteins show bidirectional particle movement in OSN cilia. Line-scan kymograms of cilia on OSNs ectopically expressing (**j**) AV-BBS1:GFP, (**k**) AV-BBS2:GFP or (**l**) AV-BBS4:GFP were generated from TIRF time-series acquisitions from native OE. (**m**) Histogram distribution of BBS4:GFP particle velocities. *n*=1,735 anterograde particles and 1,146 retrograde particles. (**n**) BBS4:mCherry associates with IFT particles. Representative kymogram was generated from a cilium of an OSN co-expressing AV-BBS4:mCherry and AV-IFT88:GFP. Scale bars, 10 μm (**a**,**c**,**e**); 2.5 μm (inset of **c**); 2.5 μm (**b**,**d**,**f**,**g**); 10 μm (**h**,**i**); 5 μm × 10 s (**j**–**l**,**n**).

Proteins homologous to those associated with human BBS participate in IFT in invertebrates^41^,^42^,^43^. Although various lines of evidence have implicated mammalian BBS proteins as being resident within pericentriolar satellites^44^, basal bodies^45^ and cilia^46^, no report has demonstrated that mammalian BBS proteins traffic with IFT. We examined constituents of the core BBSome^46^ via expression of FP-tagged BBS1, BBS2 and BBS4 in OSNs. Confocal microscopy revealed strong enrichment of BBSome proteins within OSN knobs (Fig. 5a–f), consistent with basal body localization. In addition, BBS2:GFP was readily detectable in puncta along OSN axonemes (Fig. 5c inset), whereas BBS1:GFP was discretely present in the proximal-most regions of OSN axonemes (Fig. 5b) and BBS4:GFP was occasionally detected at ciliary tips (Fig. 5g). Using TIRFm, we observed that each of the three BBSome fusion proteins undergo bidirectional particle movement along the length of OSN cilia (Fig. 5j–l and Supplementary Movie 4) and do so with velocities similar to FP-tagged IFT proteins (Fig. 5m and Table 1; *n*=2,881 BBS4:GFP-labelled particles, 143 cilia, 48 OSNs and 7 mice). We next co-expressed BBS4:mCherry and IFT88:GFP, and found that BBS4:mCherry was present in all observed IFT88:GFP-labelled particles (Fig. 5n and Supplementary Movie 5), verifying that the BBSome is in fact a constituent of the IFT particle in OSN cilia.

Assembly of the BBSome depends up the activity of a BBS–chaperonin complex containing BBS6, BBS10 and BBS12 (ref. 47). As BBS10 and BBS12 are present at or near basal bodies^48^ where the BBSome is most abundant in primary ciliated cells, we hypothesized that they may also associate with the BBSome during IFT in OSN cilia. Confocal analysis revealed BBS10:GFP localization almost exclusively in OSN knobs (Supplementary Fig. 4). Within the OSN knob, however, BBS10:GFP did not associate with all basal bodies as is typical for BBS proteins; instead, BBS10 accumulated as a single focus (Supplementary Fig. 4b–e). Remarkably, the BBS10:GFP focus was highly dynamic, displaying constant mobility (Supplementary Fig. 4f). It is enticing to speculate that BBS10 and other components of the BBS–chaperonin complex are resident within a single pericentriolar endosome that actively shuttles throughout the OSN knob to provide support to each olfactory cilium. Using TIRFm on OSNs co-expressing BBS10:GFP and BBS4:mCherry, we observed only rare, transient association of BBS10 with IFT particles (Supplementary Fig. 4g). Thus, we conclude that the major function of the BBS–chaperonin complex in BBSome assembly is performed outside of OSN cilia before incorporation of the BBSome into IFT particles.

### Peripheral membrane Arl proteins are OSN IFT cargoes

**Figure 6:**
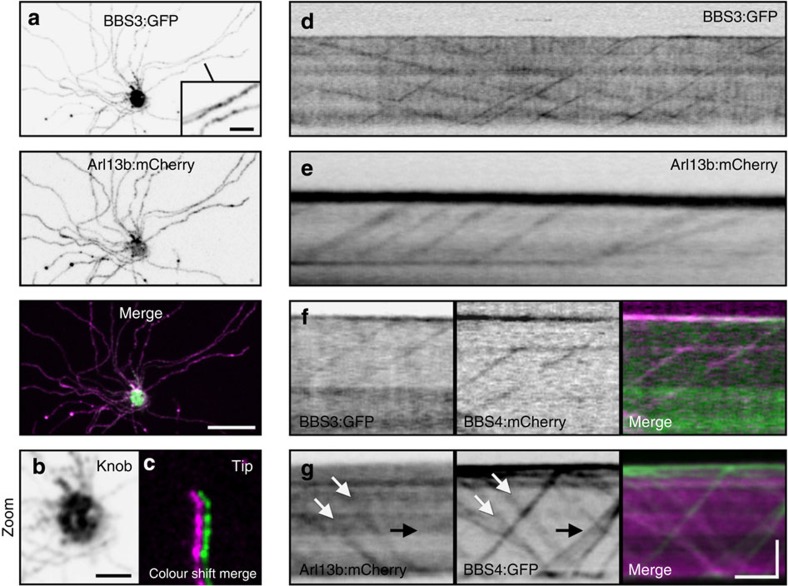
Peripheral ciliary membrane-associated Arl proteins participate in IFT. (**a**–**c**) Representative live *en face* confocal images of native OE ectopically co-expressing AV-BBS3:GFP and AV-Arl13b:mCherry. (**a**) BBS3:GFP is present along OSN ciliary axonemes. Inset shows zoomed image of two axonemes decorated with BBS3:GFP. (**b**) Zoomed image of the OSN knob showing BBS3:GFP accumulation. (**c**) Zoomed and colour-shifted image of an OSN ciliary distal tip showing co-localization of BBS3:GFP and Arl13b:mCherry. (**d**) Line-scan kymogram generated from a TIRFm time series of a BBS3:GFP AV-transduced OSN showing bidirectional movement of BBS3:GFP in an OSN cilium. (**e**) Line-scan kymogram generated from a TIRFm time series of an Arl13b:mCherry AV-transduced OSN showing particle movement of Arl13b:mCherry in an OSN cilium. (**f**) Line-scan kymogram of an OSN cilium co-expressing AV-BBS3:GFP and AV-BBS4:mCherry. (**g**) Line-scan kymogram of an OSN cilium co-expressing AV-Arl13b:mCherry and AV-BBS4:GFP. White arrows highlight a particle possessing both Arl13b:mCherry and BBS4:GFP. Black arrow indicates a group of particles that are labelled by BBS4:GFP but not Arl13b:mCherry. Scale bars, 10 μm (**a**); 2.5 μm (**a** (inset), **b**,**c**); 5 μm × 10 s (**d**–**g**).

We next examined peripheral ciliary membrane Arl proteins. Arl6 (hereafter referred to as BBS3) is required for BBSome recruitment onto membranes *in vitro*^49^ and for localization of the BBSome within mammalian cilia on cultured cells^50^. In *C. elegans*, BBS3 and Arl13b homologues move bidirectionally within cilia, suggesting they associate with IFT^41^,^51^. Similar to Arl13b:mCherry, we observed BBS3:GFP throughout the full length of OSN axonemes (Fig. 6a,c). Remarkably, both BBS3:GFP and Arl13b:mCherry showed discrete bidirectional ciliary transport when imaged by TIRFm (Fig. 6d–g and Supplementary Movie 6). To assess the stoichiometry of BBS3 and Arl13b association with IFT particles, we co-expressed each protein with FP-tagged BBS4 and performed dual colour time-lapse TIRFm. Although BBS3:GFP appeared to co-localize with all BBS4:mCherry-labelled particles (Fig. 6f), Arl13b:mCherry was clearly absent from most (69.2%) BBS4:GFP particles (*n*=340) (Fig. 6g and Supplementary Movie 7), indicating that Arl13b is an intermittent IFT cargo and not a core constituent of the IFT machinery.

### Polytopic signalling proteins rarely associate with OSN IFT

**Figure 7:**
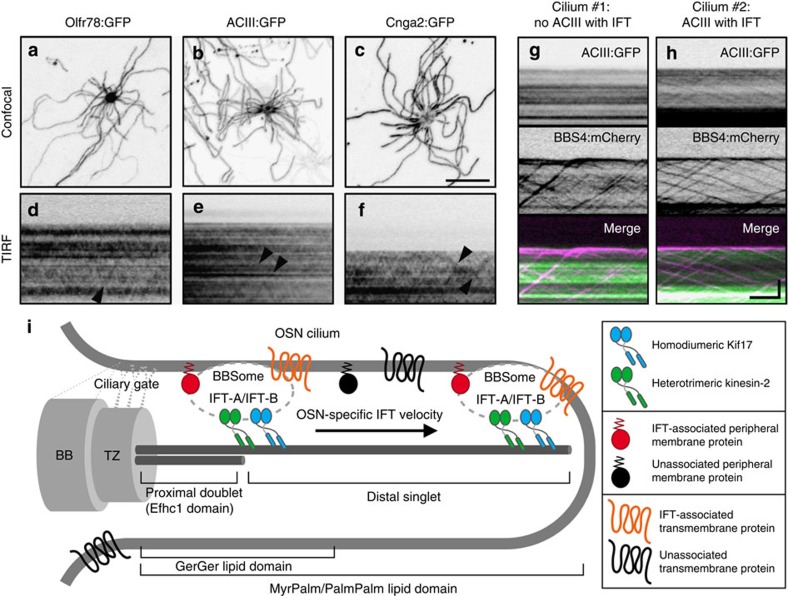
Polytopic olfactory signalling proteins have the capacity to undergo IFT. (**a**–**c**) Representative confocal images of AV-transduced OSNs expressing (**a**) Olfr78:GFP, (**b**) ACIII:GFP or (**c**) Cnga2:GFP. Each FP-tagged olfactory signalling protein was distributed along the full length of OSN cilia. (**d**–**f**) Representative time-lapse kymograms generated from cilia on AV-transduced OSNs expressing (**d**) Olfr78:GFP, (**e**) ACIII:GFP or (**f**) Cnga2:GFP. In each panel, occasional IFT-like translocation events are seen (arrowheads). (**g**,**h**) Representative time-lapse kymograms generated from two cilia on a single AV-transduced OSN co-expressing ACIII:GFP and BBS4:mCherry. In **g**, no association of ACIII with BBS4 is seen. In **h**, several BBS4-labelled IFT particles carry ACIII. Scale bars, 10 μm (**a**–**c**); 5 μm × 10 s (**d**–**h**). (**i**) Model of ciliary organization and IFT in OSNs. OSN cilia feature short (~2.5 μm) doublet proximal microtubules that transition to singlets that can span more than 100 μm. The OSN ciliary membrane gate located at or near the basal body (BB)/transition zone (TZ) complex is permissive to the ciliary entry of prenylated and acylated proteins, which differentially partition once within the OSN ciliary membrane. The mammalian OSN IFT particle comprises IFT-A, IFT-B and BBSome particle subcomplexes, and associates with both homodimeric Kif17 and heterotrimeric kinesin-2 along the full axoneme length. OSN IFT particles intermittently carry both peripheral membrane-associated proteins and polytopic olfactory signalling proteins as cargoes.

A major unanswered question is how olfactory receptors and other ciliary transmembrane proteins are trafficked within cilia. Evidence from invertebrates has demonstrated the capacity of transient receptor potential vanilloid channels to processively traffic with IFT^52^,^53^. By contrast, Ye *et al*.^16^ recently reported that in mammalian digitonin-permeablized cells, the G protein-coupled receptor SSTR3 and Smo only associate with IFT over short intervals and more often move by diffusion. To address whether mammalian transmembrane signalling proteins use IFT for ciliary trafficking within native OSNs, we expressed an FP-tagged odourant receptor, adenylate cyclase and a subunit of the olfactory cyclic nucleotide-gated ion channel (Olfr78:GFP, ACIII:GFP and CNGA2:GFP, respectively). By confocal microscopy, each FP-tagged signalling protein showed distribution throughout OSN ciliary membranes (Fig. 7a–c). Through TIRFm, we discerned brief translocation events for each FP-tagged protein (Fig. 7d–f), reminiscent of disordered SSTR3 and Smo movement in cultured cell cilia^16^. To verify whether these movements correspond to IFT particles, we co-expressed ACIII:GFP and BBS4:mCherry, and found that ACIII:GFP movements were indeed coincident with IFT trafficking (Fig. 7h and Supplementary Movie 8). Interestingly, only a fraction of all IFT particles (14.4% of 479 particles captured from 60 cilia on 9 cells) were associated with ACIII and, moreover, only a fraction of all cilia (36.7%) showed any IFT-associated ACIII translocation during the given interval of data acquisition (Fig. 7g,h). Our data indicate that although transmembrane signalling proteins can use IFT for movement within OSN cilia, processive translocation events are rare at steady state, and we predict the function of these proteins is probably not dependent on constant association with IFT.

## Discussion

Genetic anomalies disrupting IFT or BBS proteins manifest a wide array of variably penetrant disease pathologies that arise from dysfunction of cilia found on cells of various tissues. In general, the genetic loci underlying clinically distinct ciliopathies are coincident with biochemical data showing interactions between their encoded proteins and cooperation as a functional unit^54^. However, genetic and phenotypic overlap among ciliopathies predicts that some ciliary units functionally interact. In support of this notion, our data reveal for the first time the BBSome as a constituent of the mammalian olfactory IFT particle and implies that altered (but not ablated) IFT underlies BBS disease pathology. Interestingly, ciliation of the OE, but not ciliation in most other tissues, is dependent on normal BBSome function^8^,^9^,^10^, suggesting the precise role of BBS proteins in IFT will vary by cell type, thereby influencing disease penetrance in different tissues. This is reflected in lower eukaryotes where loss of BBS proteins has species-specific consequences. In *C. elegans* the BBSome is essential for IFT particle integrity and motor coordination^37^,^55^,^56^, but in *C. reinhardtii* BBS proteins are dispensable for IFT and flagellar biogenesis^34^. *C. reinhardtii* BBSome proteins associate with only a subset of IFT particles, whereas IFT particles in OSN cilia show a 1:1 ratio of BBS protein labelling. This 1:1 stoichiometry may represent a critical requirement specifically in OSNs for BBS proteins to facilitate assembly and/or maintenance of cilia via modulation of the IFT machinery. The mammalian BBSome may function similar to its role in *C. elegans* where the coupling of IFT-A and IFT-B into a complete unit is dependent on the presence of the BBS proteins. The inability of the A and B subcomplexes to form a fully functional unit would probably have major ramifications on their ability to properly maintain the remarkably long axonemes of OSN cilia. Alternatively, the BBSome itself may provide docking sites for IFT cargoes that are necessary for assembly/maintenance of OSN cilia. Although biochemical data is lacking, genetic disruption of a BBS protein combined with reduced IFT88 expression exacerbates ciliopathy phenotypes in mouse^57^ and *IFT27* mutations give rise to BBS phenotypes in humans^7^, together demonstrating a potential functional link between the mammalian BBSome and IFT. Direct analysis of IFT assembly in the context of BBS mutant OSNs should provide insight into the functional relationship between the mammalian BBSome and IFT in the future. In addition to alterations in IFT function, the loss of OSN cilia seen in BBS mutant animals may also occur as a consequence of compromised ciliary membrane delivery, a role in which the BBSome is implicated^49^. As OSN cilia require relatively large amounts of membrane to encapsulate their full length, ciliary membrane defects due to BBS mutations might have a greater adverse effect on the length of these cilia compared with cilia on other cell types.

The current model of proximal–distal kinesin-based axoneme assembly, generated from studies in *C. elegans* amphid/phasmid channel sensory cilia, states that two motor complexes cooperate to build distinct compartments of the cilium; the Kif17 homologue OSM-3 is solely responsible for distal singlet microtubule biogenesis and heterotrimeric kinesin-2 mediates IFT in cooperation with OSM-3 only along the proximal segment^20^,^37^. This model is incompatible with our data showing that Kap3a travels throughout OSN cilia. Interestingly, in *C. elegans dyf-5* MAP kinase loss-of-function mutants, the heterotrimeric kinesin-2 motor abnormally gains access to the distal segment, suggesting that the underlying microtubule architecture itself does not dictate kinesin usage^58^. Thus, a divergent phosphorylation programme may be responsible for the differential localization of heterotrimeric kinesin-2 in OSN cilia versus *C. elegans* amphid/phasmid channel cilia. We also find that Kif17 is dispensable for both OSN distal segment formation/maintenance (Fig. 3) and for sustaining anterograde velocity (Table 1). Although this data is in contrast with the ciliogenic role of OSM-3 in *C. elegans* amphid/phasmid channel cilia distal segments, it is consistent with the dispensability of OSM-3 in amphid wing `C' cell ciliogenesis^59^ and in the formation of amphid wing `B' (AWB) cell distal segments^60^. However, in AWB cilia the heterotrimeric kinesin-2 is nevertheless restricted from distal segments, highlighting a divergence between AWB and OSN cilia as well^60^. In support of our findings, disruption of Kif17 in cultured cells affects ciliary cargo trafficking without having an essential function in ciliogenesis^61^, and genetic ablation of Kif17 is apparently not inhibitory to ciliation in mice^62^. Instead, formation of OSN ciliary distal segments is probably facilitated by one or more other kinesins and our data points to heterotrimeric kinesin-2, which distinctively participates in IFT along the full length of OSN axonemes. Indeed, heterotrimeric kinesin-2 is solely responsible for proximal and distal segment formation in the cilia of *Drosophila* olfactory receptor neurons^63^. Overall, our data uncovers a unique collaboration between the heterotrimeric and homodimeric kinesin-2 motors in OSN cilia and further demonstrates how kinesin usage is diversified in different organisms and cell types.

IFT velocities significantly vary among neighbouring OSNs. This is not surprising, considering the extent of heterogeneity that defines the OE. Each OSN within the olfactory system predominantly expresses one of ~1,000 odourant receptors^64^,^65^; the OE therefore comprises subpopulations of cells that are differentially activated by various stimuli. As changing intracellular cyclic AMP and calcium influences IFT velocity^17^, we hypothesize that differential basal stimulation through environmental odours could have downstream effects to produce the cell-by-cell variation in IFT dynamics we observed. Similarly, the stochastic variation may be influenced by spatial distribution of individual OSNs within the OE or their age/maturation state.

Delineating the mechanisms of ciliary cargo trafficking, and membrane protein trafficking in particular, remains a key area of ongoing research interest. Recent data from single-molecule imaging in cultured cells suggests that individual ciliary membrane signalling receptors dynamically switch between short bursts of active transport by IFT and longer intervals of passive diffusion^16^. Likewise, clusters of flagellar membrane glycoproteins transiently associate with moving IFT particles in *C. reinhardtii*^66^. However, previous ensemble imaging has shown that populations of certain membrane proteins can associate with IFT particles over comparatively longer distances^52^,^53^. Our ensemble data showing peripheral membrane Arl proteins and transmembrane olfactory signalling proteins undergoing bouts of processive translocation in OSN cilia clearly demonstrates a capacity for these cargoes to use IFT in their trafficking in a native mammalian setting. However, the associations between IFT and the cargo proteins analysed here are intermittent and infrequent among OSN cilia and particles within cilia, suggesting that continuous association between cargo and particle is not essential to the normal function of these proteins.

Overall, from this work we have deconstructed mammalian OSN cilia axoneme and membrane organization, the composition and dynamic properties of OSN IFT and the degree to which IFT contributes to peripheral and transmembrane protein trafficking. Our data establish for the first time BBS protein participation in vertebrate IFT and provide novel insight into how kinesin motors are used in OSN cilia (Fig. 7i). Although invertebrate studies have informed our understanding of the players involved in ciliary transport, our findings highlight divergent qualities of mammalian IFT. In addition, the unique aspects of OSN IFT versus other mammalian cell types (Table 1) underscores the necessity to ascertain how cellular specification of the IFT machinery affects tissue-specific ciliopathy disease penetrance.

## Methods

### Mice

CD1 and GFP-CETN2 (Jackson Laboratory) mice were housed at the University of Florida. All procedures were approved by the University Committee for the Use and Care of Animals. Unless otherwise stated, two to three mice were used in each experimental condition.

### Scanning electron microscopy

After deep anaesthesia and transcardial perfusion with 2% glutaraldehyde, 0.15 M cacodylate in water, olfactory tissue was processed using the OTOTO protocol. Analysis was performed on an Amray (Drogheda, Ireland) 1910FE field emission scanning electron microscope at 5 kV and recorded digitally with Semicaps software.

### Constructs

Plasmids containing complementary DNA inserts used in this study were generously provided as follows: Nphp4:mCherry and IFT88 (B.K.Y., UAB); GFP:Efhc1 (K.Y., RIKEN); Arl13b (T.C., Emory); IFT122:GFP (J.E., UGA); Kap3a (T.S., Johns Hopkins); Olfr78 (J.P., Johns Hopkins); ACIII (R.R., Johns Hopkins); BBS1 (V.C.S., Iowa); and BBS2, BBS3, BBS4 and BBS10 (K.M., Ohio State). Dync2li1 was amplified from cDNA generated from mouse whole eye RNA. Lipid-anchored GFP constructs were generated by annealing oligonucleotides encoding the 13 amino-terminal residues from Lyn kinase (MyrPalm), the 20 N-terminal residues from GAP43 (PalmPalm) and the 9 carboxy-terminal residues from the guanine triphosphatase Rho (GerGer) into the KpnI and AgeI sites of pEGFP-N1 (Clontech). All cloning primer sequences are available on request.

### Analysis of ectopic expression in mouse OSNs

For expression in native tissue, recombinant GFP-, mCherry- or mCitrine-flanked cDNAs were inserted into the adenoviral vector pAD/V5/−dest and virus was propagated using the ViraPower protocol (Invitrogen). AV particles were isolated with the Virapur Adenovirus mini purification Virakit (Virapur, San Diego, CA) and dialysed in 2.5% glycerol, 25 mM NaCl and 20 mM Tris-HCl, pH 8.0. Twenty-microlitre volumes of AV-containing constructs of interest were intranasally administered to the OE of pups at P7, P8 and P9. Transduced animals were killed for analysis on or after day P19.

For immunostaining, mice were deeply anaesthetized and then cardiac perfused with 4% paraformaldehyde. The lower jaw, teeth and hair were removed from snouts before postfixation in 4% paraformaldehyde for 2 h at 4 °C. Snouts were then decalcified in 0.5 M EDTA/ × PBS overnight, cryoprotected in 10%, 20% and 30% sucrose for 1 h, 1 h and overnight, respectively, at 4 °C, and embedded in optimal cutting temperature compound (Tissue-Tek). Coronal cryosections were sliced at a thickness of 10–12 μm and mounted onto Superfrost Plus slides (Fisher Scientific). For immunostaining, cryosections were permeabilized and blocked with 0.3% Triton X-100 and 2% goat serum in PBS for 30 min. Samples were incubated with acetylated α-tubulin antibody (clone 6–11 B-1, Sigma T6793) diluted 1:1,000 in 2% goat serum in PBS for 1 h at room temperature and washed three times with PBS. Cryosections were incubated in fluorescent-conjugated secondary antibodies for 1 h, washed three times with PBS and mounted using Prolong Gold (Invitrogen). Fixed tissue imaging was performed on an Olympus Fluoview 500 confocal microscope equipped with a 405-nm laser diode with a 430- to 460-nm band-pass filter, a 488-nm laser with a 505- to 525-nm band-pass filter, a 543-nm laser with a 560-nm long-pass filter and a 633-nm laser with a 660-nm long-pass filter; original magnification × 60, 1.40 numerical aperture (NA) or × 100,1.35 NA oil objectives were used.

For confocal *en face* imaging of transduced OSNs, animals were anaesthetized with CO_2_, rapidly decapitated, split along the cranial midline and the OE was dissected out in PBS. Turbinates were placed in a tissue chamber and imaged on a Nikon TiE-PFS-A1R confocal microscope equipped with a 488-nm laser diode with a 510- to 560-nm band-pass filter and 561 laser with a 575- to 625-nm band-pass filter. A CFI Apo Lamda S × 60, 1.4 NA objective was used.

For *en face* TIRFm imaging of transduced OSNs, animals were anaesthetized with CO_2_, rapidly decapitated, split along the cranial midline and further prepared in artificial cerebrospinal fluid (124 mM NaCl, 3 mM KCl, 1 mM MgCl_2_, 2 mM CaCl_2_, 1.25 mM NaH_2_PO_4_, 26 mM NaHCO_3_, 25 mM glucose) bubbled with 5% CO_2_/95% O_2_ for 10 min before use. The rostral- and caudal-most portions of one hemisphere were removed, leaving the OE and olfactory bulb intact in the skull. The tissue was placed face down in the imaging chamber, bathed in 35 °C artificial cerebrospinal fluid and held in place with mesh netting. TIRFm time series were captured at 200 ms exposure for 2–3 min on a Nikon Eclipse Ti-E/B inverted microscope equipped with a × 100 CFI APO TIRF 1.49 NA, × 1.5 tube lens, ZT488/561rpc dichroic, ZET488/561 × excitation filter, ZET488/561m-TRF emission filter (Chroma Technology) and an electron-multiplying charge-coupled device camera (iXon X3 DU897, Andor Technology). The 488-nm line of a fibre-coupled diode laser at incident power of 2 mw was used to illuminate a circular region of ~60 μm in diameter for capturing video sequences at 5–10 Hz. ImageJ was used to generate line-scan kymograms for measuring particle velocities from imported time series. Velocity was calculated with the following formula: *tan*(α*π/180)*b/*c* where *α* is angle, *b* is calibration in μm per pixel and *c* is exposure time per frame.

### Statistical analysis

As our IFT velocity data set significantly violated the constant variance assumption of analysis of variance, we employed a non-parametric permutation test to assess the variability of IFT rates on the cell and population level using the following formula, to obtain test statistics:


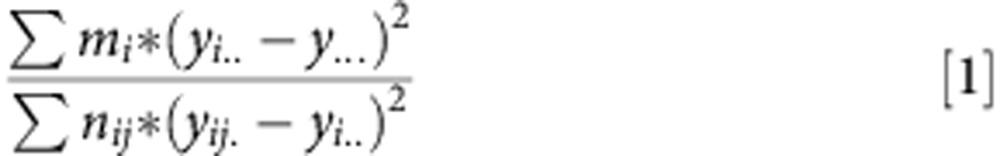


The numerator reflects the variance between the cells and the denominator reflects the variance within the cells (that is, between the cilia); *i* is the cell index, *j* is the cilia index. The designating observations are denoted by *y*_*ijk*_ where *k* is the IFT particle index. The notations *y*_*ij.*_, *y*_*i..*_ and *y*... denote the average values of observations in a cilium, in a cell or in the entire population, respectively. Number of observations in a cilium were given as *n*_*ij*_ and number of observations in a cell were given as *m*_*i*_. Calculation of anterograde and retrograde observations gave statistics of 14.23 and 10.12, respectively. Next, the anterograde and retrograde observations were permuted and randomly shuffled across cells 20,000 times and the test statistics were calculated for each permutation. The *P*-values were calculated to be the percentages of the test statistics obtained from permutated data sets falling below the test statistics obtained from the observed data. For both the anterograde and retrograde data, the *P*-values were <0.0001, demonstrating that variations in IFT velocities among cells are significantly greater than variations among cilia in each cell.

## Author contributions

C.L.W, J.C.M., P.M.J. and J.R.M. designed experiments. C.L.W., J.C.M., S.R.N. and P.M.J. performed experiments. C.L.W., J.C.M., P.M.J. and L.Z. generated reagents. Q.P. performed biostatistical analysis. K.V. provided equipment and reagents. C.L.W. and J.R.M. wrote the manuscript, with all authors providing input. C.L.W. made illustrations and generated figures. J.R.M. directed the project.

## Additional information

**How to cite this article:** Williams, C. L. *et al*. Direct evidence for BBSome-associated intraflagellar transport reveals distinct properties of native mammalian cilia. *Nat. Commun.* 5:5813 doi: 10.1038/ncomms6813 (2014).

## Supplementary Material

Supplementary Figures and Supplementary Methods Supplementary Figures 1-4 and Supplementary Methods

Supplementary Movie 1 Processive bidirectional trafficking of kinesin and dynein complex proteins in olfactory sensory cilia.

Supplementary Movie 2 Disruption of Kif17 does not affect kinesin-2 trafficking to distal tips of olfactory sensory cilia.

Supplementary Movie 3 Processive bidirectional trafficking of IFT-A and IFT-B subcomplex proteins in olfactory sensory cilia.

Supplementary Movie 4 Processive bidirectional trafficking of BBSome proteins in olfactory sensory cilia.

Supplementary Movie 5 Co-movement of BBS4 and IFT88 in olfactory sensory cilia.

Supplementary Movie 6 Processive bidirectional trafficking of peripheral membrane-associated Arl proteins in olfactory sensory cilia

Supplementary Movie 7 Co-movement of Arl13b and BBS4 in olfactory sensory cilia.

Supplementary Movie 8 Co-movement of ACIII and BBS4 in olfactory sensory cilia

## Figures and Tables

**Table 1:** Comparison of IFT velocities across species and cell types.

**Species/cell type**	**Marker**	**Length (μm)**	**Anterograde (μm s**^−1^**)**	**Retrograde (μm s**^−1^**)**	**Citation**
*C. reinhardtii*	DIC	12	1.8	3.1	Iomini *et al*.^67^
*C. reinhardtii*	IFT20:GFP	12	2.1	2.5	Lechtreck *et al*.^43^
*C. reinhardtii*	IFT27:GFP	2–4	~1.6	nd	Engel *et al*.^68^
*C. reinhardtii*	IFT27:GFP	6–8	~2.0	nd	Engel *et al*.^68^
*C. reinhardtii*	IFT27:GFP	10+	~2.3	nd	Engel *et al*.^68^
*Trypanosoma brucei* (27 °C)	GFP:IFT52, GFP:DHC2.1	22.3	2.4 (1.5)*	5.6	Buisson *et al*.^39^
*T. brucei* (35 °C)	GFP:IFT52, GFP:DHC2.1	22.3	3.2 (2.2)*	7.4	Buisson *et al*.^39^
*C. elegans*/Amphid-middle	OSM-6:GFP	4	0.68	1.2	Snow *et al*.^37^
*C. elegans*/Amphid-distal	OSM-6:GFP	2.5	1.3	1.1	Snow *et al*.^37^
*Mus musculus*/LLC-PK1	IFT20:GFP	~6	~0.6	~0.7	Follit *et al*.^14^
*M. musculus*/IMCD3	IFT88:YFP	6	0.38	0.56	Tran *et al*.^13^
*M. musculus*/MEF	IFT88:GFP	~3	1.09	0.72	He *et al*.^18^
*M. musculus*/OSN-distal	IFT122:GFP	2.5–100+	0.22	0.14	Current work
*M. musculus*/OSN-distal	IFT88:GFP	2.5–100+	0.23	0.14	Current work
*M. musculus*/OSN-distal	Kif17:mCitrine	2.5–100+	0.23	nd	Current work
*M. musculus*/OSN-distal	Kif17DN:GFP	2.5–100+	0.23	nd	Current work
*M. musculus*/OSN-distal	BBS4:GFP	2.5–100+	0.24	0.17	Current work

Nd, not determined.

^*^Subset of slow particle velocities.
