# Supplementary material for: Direct evidence for BBSome-associated intraflagellar transport reveals distinct properties of native mammalian cilia
Source: Nat Commun. 2014 Dec 15;5:5813. doi: 10.1038/ncomms6813 (PMC4284812; doi:10.1038/ncomms6813)
Supplement: Supplementary Figures and Supplementary Methods — Supplementary Figures 1-4 and Supplementary Methods [file ncomms6813-s1.pdf]

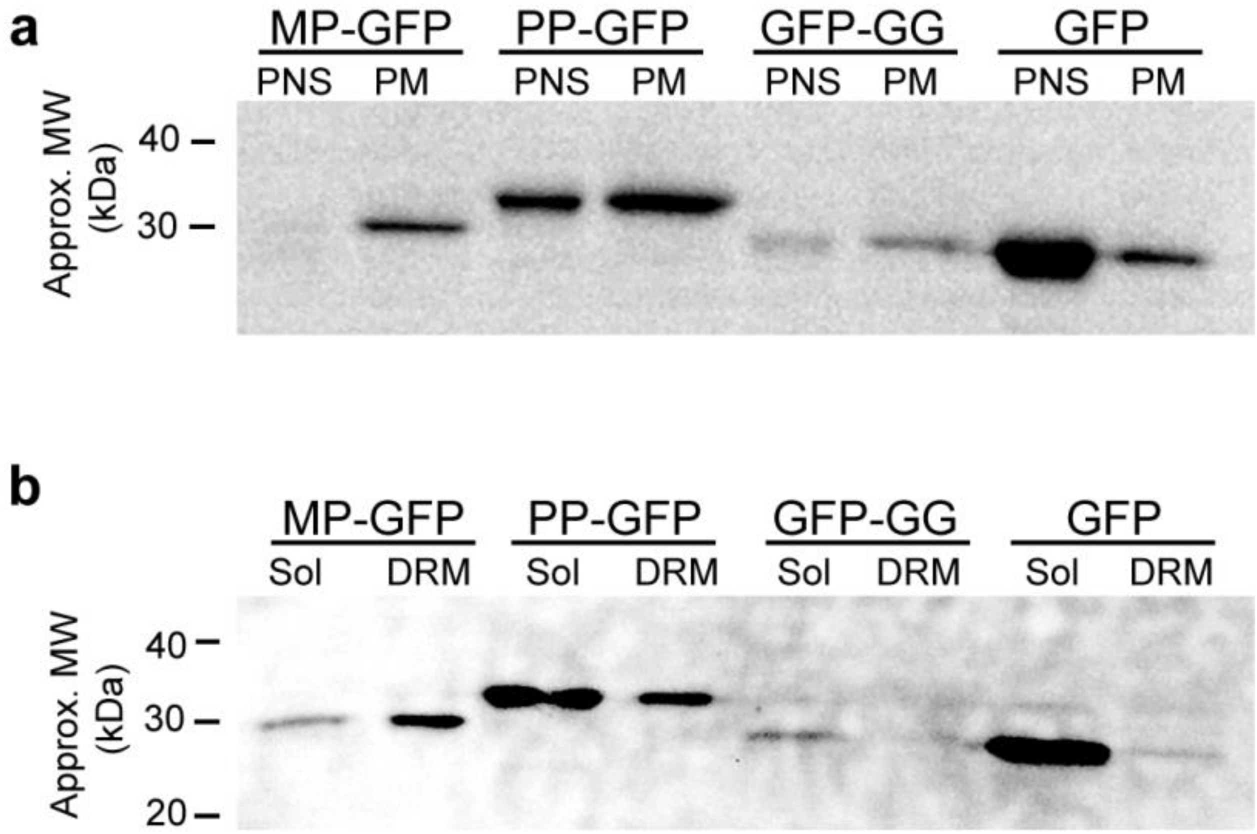

Supplementary Figure 1. Differential membrane partitioning of lipid-modified GFPs. HEK293 cells expressing GFP, MyrPalm-GFP, PalmPalm-GFP, or GFP-GerGer were (a) lysed in the absence of detergent to isolate plasma membrane (PM) and post-nuclear supernatant (PNS) fractions, or were (b) lysed in the presence of 1% Triton X-100 to isolate soluble protein (Sol) and detergent-resistant membrane (DRM) fractions. Fractions were run on SDS-PAGE and immunoblotted with an anti-GFP antibody. MyrPalm-GFP (MP-GFP) and PalmPalm-GFP (PP-GFP) were found in the DRM fraction, whereas GFP-GerGer (GFP-GG) and soluble GFP were excluded from the DRMs and localized exclusively to the soluble fraction.

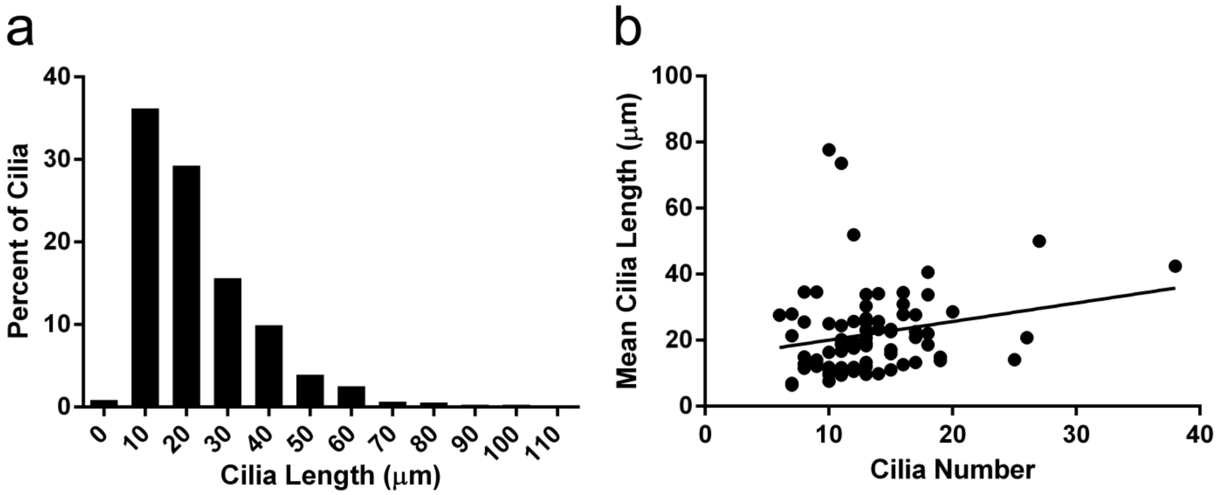

Supplementary Figure 2. Measurements of OSN cilia lengths. (a) Histogram distribution of OSN cilia lengths measured from live *en face* confocal images of OSNs ectopically expressing MyrPalm-GFP as a cilium axoneme/membrane marker. Overall mean cilium length was 22.0+/-12.8 μm. (b) Plot of cilia number versus mean cilia length per OSN. Pearson's R_2 coefficient = 0.04829. Data were collected from 81 OSNs and 1083 cilia.

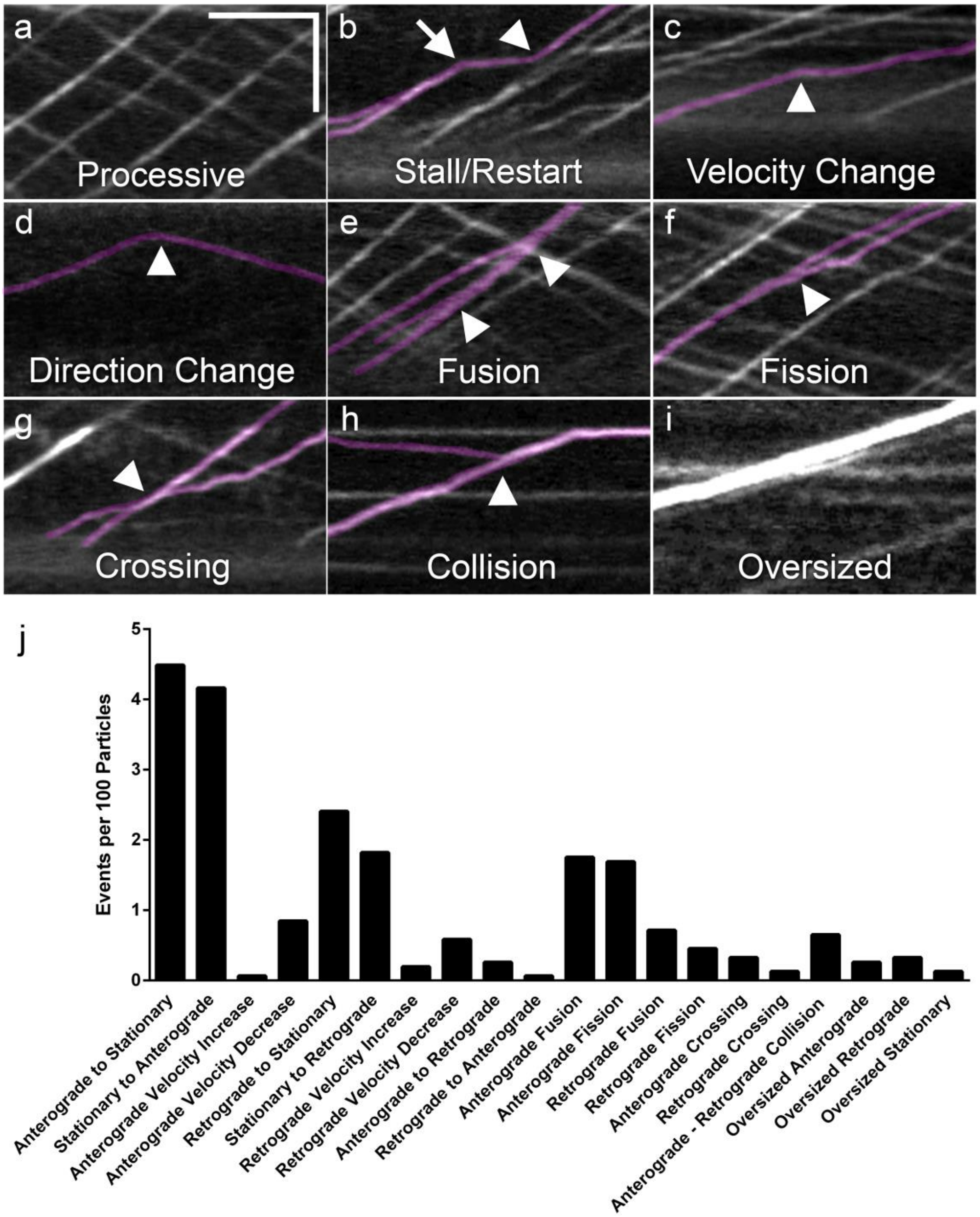

Supplementary Figure 3. Rare OSN IFT particle behaviours. Line scan kymograms of GFP-labeled IFT particles showing rare events that deviate from (top, left) processive motion. Some particles of interest are pseudocolored purple for clarity. Each panel is oriented such that the distal tip of the cilium depicted would be located above the image. (a) Processive anterograde and retrograde particle motion. (b) An anterograde particle stalls (arrow) and later restarts (arrowhead). (c) An anterograde particle undergoes a velocity change (arrowhead). (d) An anterograde particle prematurely reverses direction. (e) Two anterograde particles fuse with and adopt the faster velocity of a third anterograde particle. (f) An anterograde particle undergoes fission into two particles. (g) A fast anterograde overtakes a slower anterograde particle without any apparent interaction. (h) A retrograde particle collides and fuses with an anterograde particle, which then proceeds to the ciliary distal tip. (i) An oversized anterograde particle. (j) Quantification of rare IFT events. n = 1538 IFT88:GFP- or Kif17:mCitrine-labeled particles captured from 93 cilia. Scale in (a-i), 5 μm x 10 s.

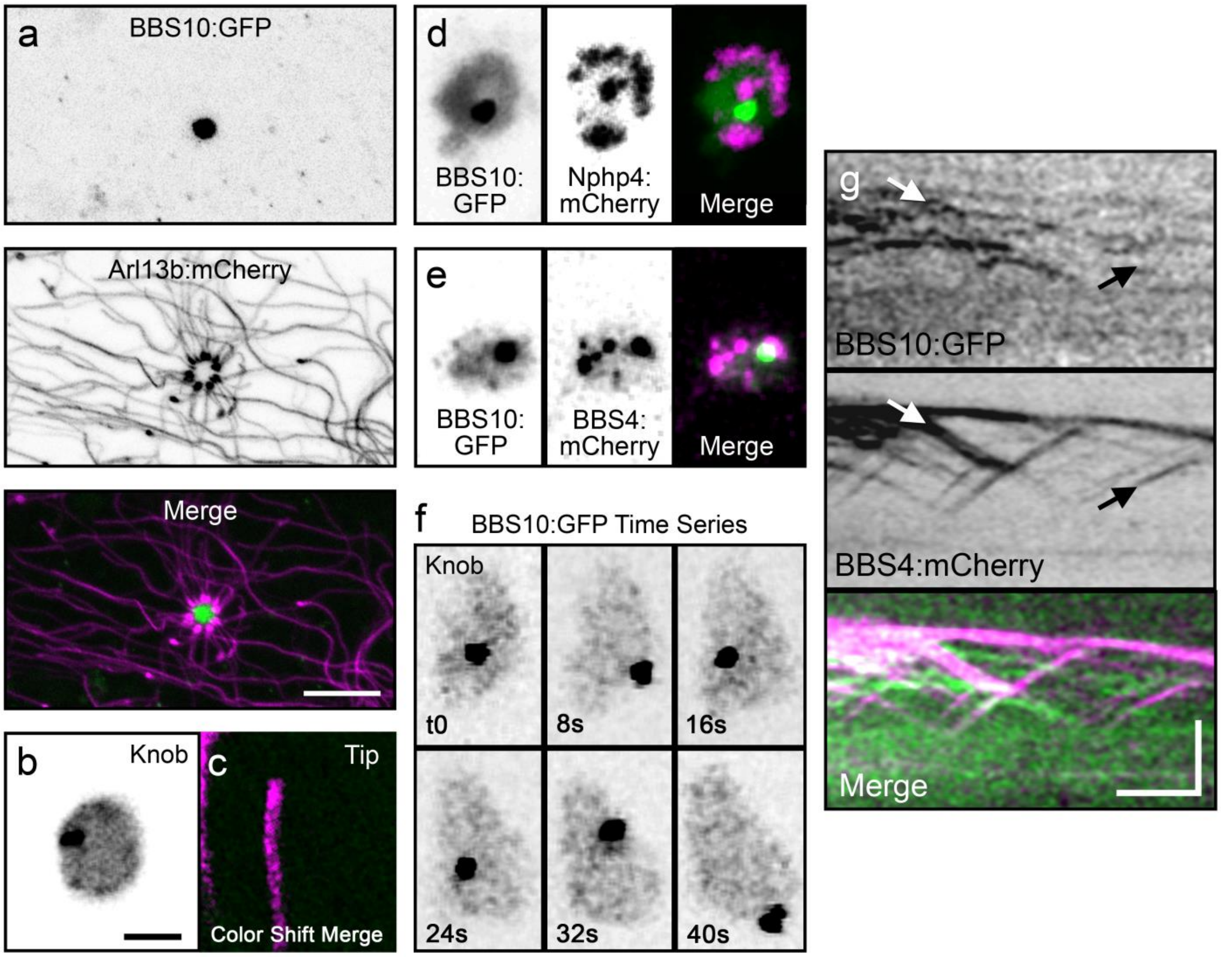

Supplementary Figure 4. BBS10 is predominantly associated with a mobile compartment in the OSN knob. (a-c) Representative live *en face* confocal images of native AV-transduced OE co-expressing BBS10:GFP and Arl13b:mCherry showing BBS10:GFP accumulation in a single focus within the OSN knob (b) and no apparent signal within OSN cilia (c). (d and e) BBS10:GFP occupies a single focus within the OSN knob, as opposed to Nphp4:mCherry and BBS4:mCherry, which decorate transition zones and basal bodies, respectively. (f) Time series showing dynamic mobility of the BBS10:GFP focus within the OSN knob. (g) Line scan kymogram generated from a cilium of an AV-transduced OSN co-expressing BBS10:GFP and BBS4:mCherry. White arrow indicates a particle that is labeled by both BBS10:GFP and BBS4:mCherry. Black arrow highlights a particle that is labeled by BBS4:mCherry but not BBS10:GFP. Scale of (a), 10 µm; (b-f), 2.5 µm; (g), 5 µm x 10 s.

Supplementary Methods

Membrane partitioning of lipid anchored-GFP constructs

HEK293 cells were transfected using Lipofectamine 2000 (Invitrogen) with the lipid anchored-GFP constructs. Cells were scraped in 25mM MES, 150mM NaCl with protease inhibitors at 4ºC, homogenized in a dounce, and then passed through a 25 gauge needle 30 times. The homogenate was centrifuged at 1000 g for 10 min at 4ºC to pellet nuclei. The supernatant was removed and centrifuged at 16000g for 1 hr at 4ºC to generate a post-nuclear supernatant (PNS) containing soluble proteins. The pellet of cellular membranes (PM) was resuspended in an equal volume of lysis buffer. To generate detergent-resistant membranes, cells were homogenized in the presence of Triton X-100. The supernatant from the 16000g, 1 hr spin contained the soluble proteins whereas the pellet contained the detergent resistant membranes. Following SDS-PAGE and transfer to nitrocellulose, samples were probed with mouse monoclonal anti-GFP (Clontech, JL-8, #632380) at 1:1000 dilution then with HRP-conjugated goat anti-mouse IgG (Invitrogen) at 1:5000 dilution.
